# *Ginkgo biloba* Extract for Patients with Early Diabetic Nephropathy: A Systematic Review

**DOI:** 10.1155/2013/689142

**Published:** 2013-02-24

**Authors:** Lei Zhang, Wei Mao, Xinfeng Guo, Yifan Wu, Chuang Li, Zhaoyu Lu, Guobin Su, Xiaoyan Li, Zhuangzhu Liu, Rong Guo, Xina Jie, Zehuai Wen, Xusheng Liu

**Affiliations:** ^1^Nephropathy Department, Guangdong Provincial Hospital of Chinese Medicine, Guangzhou 510120, China; ^2^Key Unit of Methodology in Clinical Research, Guangdong Provincial Hospital of Chinese Medicine, Guangzhou 510120, China; ^3^The Second Clinical Medical School, Guangzhou University of Chinese Medicine, Guangzhou 510405, China; ^4^National Center for Design Measurement and Evaluation in Clinical Research, Guangzhou University of Chinese Medicine, Guangzhou 510405, China

## Abstract

*Objectives*. To evaluate the effectiveness and safety of a *Ginkgo biloba* extract for patients with early diabetic nephropathy. *Methods*. Randomised controlled trials (RCTs) conducted on adults with early diabetic nephropathy which used *Gingko biloba* extract were included. The major databases were searched, and manufacturers of *Gingko biloba* products were contacted for information on any published or unpublished studies. Two authors independently extracted the data from the included studies. Data analysis was conducted using Review Manager 5.0 software. *Results*. Sixteen RCTs were included. *Ginkgo biloba* extract decreased the urinary albumin excretion rate (UAER), fasting blood glucose (FBG), serum creatinine (SCR), and blood urea nitrogen (BUN). The extract also improved hemorheology. The methodological quality in the included studies was low. The explicit generation of the allocation sequence was described in only 6 trials. None of the included trials were confirmed to use blinding. Three studies had observed adverse events. One study using angiotensin-converting enzyme inhibitor (ACEi) reported mild cough in both groups. No serious adverse effects were reported. *Conclusions*. *Gingko biloba* extract is a valuable drug which has prospect in treating early diabetic nephropathy, especially with high UAER baseline level. The safety for early diabetic nephropathy is uncertain. Long-term, double-blinded RCTs with large sample sizes are still needed to provide stronger evidence.

## 1. Introduction

Diabetic nephropathy is one of the most serious complications in patients with diabetes. It is now the major leading cause of end-stage renal failure throughout the world [1].

The natural history of diabetic nephropathy includes five stages, starting with initial hyperfunction and hypertrophy at diagnosis, followed by increased glomerular filtration rate (GFR) with normal albumin excretion, incipient diabetic nephropathy (characterised by microalbuminuria), then overt clinical nephropathy leading to progressive renal failure, followed by end-stage renal disease (ESRD) with uremia [2].

Throughout the process, the relentless decline in renal function that starts at stage 4 (overt clinical nephropathy) with a mean GFR fall rate of approximately 1 mL/min^2^ is a prominent feature in patients with diabetic nephropathy. When diabetic nephropathy develops into ESRD, renal replacement therapy (RRT) is required for survival. This has a major societal impact because of the enormous financial burden on patients and governments. Moreover, the survival of patients with diabetes undergoing dialysis is much worse than that of nondiabetic patients [3, 4]. The UK Prospective Diabetes Study (UKPDS 64) [5] showed that the annual death rate of patients with elevated plasma creatinine or who were undergoing RRT was 19.2%, whereas that of patients with microalbuminuria was 3.0%, and their condition did not progress to RRT even after a year. Therefore, early therapeutic intervention in patients with diabetic nephropathy is essential.

The use of an ACEi or an angiotensin II receptor blocker (ARB) is now a component of standard therapy for patients with diabetic nephropathy, along with the control of glucose, lipids, and blood pressure. However, many patients continue to show progressive kidney damage. One study [6] that included a US population showed that among patients with diabetic nephropathy, the effect of ACEi/ARB in slowing progression to ESRD was not optimal. There is still the need to develop new therapies to improve diabetic nephropathy treatment.

At present, many botanical medicines are applied as complementary therapy for diabetic nephropathy. *Gingko biloba* extract is one of the few Chinese herbal preparations that is recognised by the international medical community [7]. It is derived from the leaves of *Gingko biloba*, the only surviving species of the Ginkgoaceae family of tree. Now it is one of the most popular over-the-counter herbal dietary supplements in the world. A standardised *Gingko biloba* extract contains 22%–27% flavone glycosides and 5%–7% terpenes [8]. Studies have shown that patients with type 2 diabetes might benefit from ingesting *Gingko biloba *extract to improve platelet function, alter platelet-vessel wall interactions [9], and reduce malondialdehyde levels in platelets [10]. 

Reduced GFR and increased albuminuria are independent risk factors for diabetic nephropathy [11]. In China, *Gingko biloba *extract has been used widely as a supplement to improve albuminuria and kidney function during the early stage (characterised by microalbuminuria) of diabetic nephropathy. Although the mechanisms underlying the pathogenesis of diabetic nephropathy are not completely understood, oxidative stress and cytokines are important factors of disease progression. Many *in vitro* and *in vivo* experiments have indicated that *Gingko biloba *extract can reduce relative total superoxide dismutase activity [12, 13] after adjusting for the expression of cytokines [14] in patients with diabetic nephropathy.

Many clinical trials have been conducted to assess the effectiveness and safety of *Gingko biloba* extract on early diabetic nephropathy, particularly in China. However, there is still no compelling evidence of the effectiveness and safety of *Gingko biloba* extract for early diabetic nephropathy, and a paper remains to be conducted.

The aim of this paper was to summarise the evidence on the effectiveness and safety of *Gingko biloba* extract for early diabetic nephropathy and to provide the best available evidence for clinical practice and further research on diabetic nephropathy treatment.

## 2. Materials and Methods

### 2.1. Criteria for Considering Studies for this Review

We included all RCTs conducted on adults (≥18 years) with early diabetic nephropathy, according to Mogensen stage III [2] (incipient urinary albumin excretion rate (UAER) of 20–200 *μ*g/min), and that used *Gingko biloba* extract, irrespective of blinding, publication status, or language.

*Ginkgo biloba* extract was added to the intervention group. All groups in the randomised trial received the same conventional treatment including glucose, lipid, and blood pressure control. The extract combined with ACEi/ARB was compared to ACEi/ARB alone. All of the included studies used standardised *Gingko biloba* extract containing 24% flavone glycosides and 6% terpenes, irrespective of dosage or form.

The primary outcome measurement was the incipient urinary albumin excretion rate (UAER) at the end of the study. Secondary outcomes were SCR, BUN, FBG, postprandial blood glucose (PBG), hemorrheology indices, and adverse reactions.

### 2.2. Search Methods for Identifying the Studies

Our search process included two steps. First, we searched all clinical trials and reviews regarding complementary and alternative treatment for diabetic nephropathy. Then we screened the clinical trials or reviews that only considered the effect of *Gingko biloba* extract on patients with early diabetic nephropathy.

We searched the following databases: PubMed (from January 1966 to September 2010), EMBASE (from January 1985 to September 2010), Cochrane library, ClinicalTrials.gov, the Chinese Biomedical Medical Database (CBM) (from 1979 to September 2010), VIP medicine information system (VMIS) (from 1989 to September 2010), China National Knowledge Infrastructure (CNKI) Database (from 1994 to September 2010), Wanfang Medicine Online (from 1998 to September 2010), and Traditional Chinese Medicine (TCM) online. More details on the search strategy are described in the Appendix.

We checked the reference lists of all acquired articles and called authors to ask for unpublished studies. In addition, we contacted manufacturers of *Gingko biloba* products to ask for information about any published and unpublished studies. We did not apply any language restrictions.

### 2.3. Data Collection

Two authors (L. Zhang and W. Mao) independently assessed the title or abstract of each record to select potential eligible studies. Full articles were retrieved for further assessment if they were graded as included or unclear. Then they independently assessed the full articles to decide which ones were to be included. Differences were resolved by a third author ( Z. H. Wen).

Two authors (L. Zhang and Z. Z. Liu) independently extracted the data using a self-developed data-extraction form, which included the following data.

1. General information: first author, published/unpublished, publication year, and location.
2. Trial design: comparison groups, method of randomisation, allocation concealment, and blinding (participants, intervention administrators, and outcome assessors).
3. Participants: disease or condition, diagnostic criteria, inclusion and exclusion criteria, total number and number in comparison groups, and baseline characteristics.
4. Interventions: treatment duration, the name and form of medication, the composition or ingredients, manufacture and quality control, and dose and administration.
5. Outcomes: outcome measures used, adverse events, author conclusions.
6. Followup: length of follow-up, any results of follow-up, reason and number of dropouts and withdrawals, and method of analysis.

### 2.4. Quality Assessment

Two authors (L. Zhang and W. Mao) independently assessed the selected trials for methodological quality using the Cochrane Collaboration tool for assessing risk of bias [31]. Trials were assessed with respect to sequence generation, allocation concealment, blinding, incomplete outcome data, selective outcome reporting, and other sources of bias. We called the authors of all of the selected trials to confirm the information of the above six domains, whether they were described in the articles or not.

We resolved discrepancies by discussion. Sometimes we consulted authors Z. H. Wen and X. F. Guo to make the final decision.

### 2.5. Measures of Treatment Effect

To measure the effects of treatments, we considered the primary and secondary outcomes detailed above. For continuous data, weighted mean differences between groups and its 95% confidence intervals were calculated when the same measurement scale was used [32].

### 2.6. Missing Data

Not all of the trials provided difference of means and its standard deviation (SD) before and after treatment in both groups. The SD was calculated using the following formula [33]:

(1) SDE,change=SDE,baseline2+SDE,final2−(2×Corr×SDE,baseline×SDE,final).

We assumed the value of the correlation coefficient (Corr) to be 0.5 between before and after treatment.

### 2.7. Assessment of Heterogeneity

We used a chi-square test to test heterogeneity and set the significance level at 0.1, in view of its low power. We also used the *I* ^2^ statistic to quantify heterogeneity. The *I* ^2^ statistic describes the percentage of variability in effect estimates that is due to heterogeneity rather than sampling error. A value >50% may indicate substantial heterogeneity.

### 2.8. Assessment of Publication Bias

We investigated publication biases of studies that included more than five trials [34] using the funnel plot.

### 2.9. Data Analysis

Data analysis was conducted using Review Manager 5.0 software, and STATA 11.0 SE. A meta-analysis was conducted on the primary and secondary outcomes mentioned above to summarise and to compare the efficacy of treatment with *Gingko biloba* extract to that of control intervention. We pooled the data using a fixed-effects model, and a randomised-effect model was used if heterogeneity was significant. The role of different baseline data was tested by metaregression analysis. In addition, we performed subgroup analyses according to different interventions, if a sufficient number of RCTs was found.

## 3. Results

### 3.1. Description of Studies

#### 3.1.1. Included Studies

**Figure 1:**
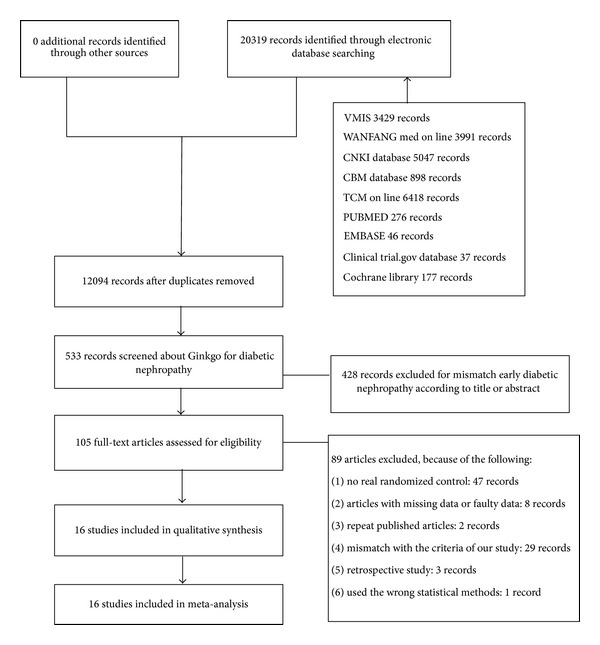
Flowchart detailing study selection.

A total of 16 RCTs, all published trials that were conducted in China, were included. For details of studies selection and included studies see Figure 1 and Table 2.We contacted all authors of trials who did not report a randomised method. However, six authors could describe the process clearly. Five trials were published between 2000 and 2005 [21, 22, 24, 26, 27], and the remaining 11 trials were published between 2006 and 2010.

The 16 included trials involved 1099 participants. Five of these studies compared ACEi/ARB alone to *Gingko biloba *extract combined with ACEi/ARB in patients with early diabetic nephropathy [15–17, 19, 28]. The others compared conventional treatment to the effect of *Gingko biloba *extract added to conventional treatment. The number of participants included in each trial varied between 45 and 112. The majority of trials included about 60 participants, and two trials included more than 100 patients [16, 24]. The mean age of the participants in the treatment groups varied between 36.7 and 66.2 years, whereas that in the control group was between 37.1 to 68.2 years. The remaining six trials had a mean age of about 50 years [17, 19, 23, 24, 28, 30]. All trials included male and female participants.

#### 3.1.2. Inclusion Criteria

Patients in all 16 trials had early diabetic nephropathy in Mogensen stage III [2]. Diabetic nephropathy was diagnosed according to the diagnostic criteria of diabetes published by WHO in 2000 for two trials [25, 29], WHO 1999 for eight trials [15, 16, 18, 19, 21, 24, 26, 28], WHO 1998 for one trial [22], and WHO 1997 for one trial [27]. Two trials [16–20, 23, 25, 28–30] used both WHO 1985 and The Americans with Disabilities Act (ADA) 1997, and two trials [17, 30] did not describe their diagnostic criteria.

#### 3.1.3. Excluded Criteria

The exclusion criteria were slightly variable among trials. In all studies, patients with other reasons for microalbuminuria, such as urinary infection, heart failure, primary hypertension, diabetic ketoacidosis, or cancer, were excluded.

#### 3.1.4. Dosage and Treatment Duration

No dose-related restrictions were included in the trials in this paper. One [29], another [22], and thirteen [15–19, 21, 23–28, 30] trials compared 10 mL (35 mg), 15 mL (52.5 mg), and 20 mL (70 mg) injections of* Gingko biloba *extract daily, respectively, versus a placebo. The remaining trial [20] used two oral *Gingko biloba* tablets (10 mg per tablet) three times per day. The treatment periods were from 14 to 60 days.

#### 3.1.5. Outcome Measurements (Table 3)

The primary outcome measurement, UAER, was mentioned in 15 trials. Regarding the secondary outcomes, 11 trials included FBG data [15, 16, 18, 20–24, 26–28], 10 trials included SCR data [15–17, 19–21, 23, 24, 26, 28], 7 trials presented BUN [16, 17, 21, 23, 24, 26, 28], 4 trials presented PBG data [21, 23, 24, 26], 3 trials presented whole blood viscosity [22, 24, 30], and plasma viscosity data [22, 23, 30]. In addition, three trials reported adverse reactions to the medication and to the placebo [19, 22, 23].

#### 3.1.6. Excluded Studies

Initially, 105 trials were prepared to be included in our study. However, after we read the articles and called the authors, 89 trials were excluded due to the following reasons. Twenty-eight trials did not conform to the literature inclusion criteria after reading the entire article and examining the data. Outcome measure data were missing or unclear in eight articles. Two articles had been published in different journals with the same data. Three trials were retrospective studies, and one trial used improper statistical methods. Finally, 48 trials were excluded after we called the authors and confirmed that randomisation was not used.

#### 3.1.7. Assessing Risk of Bias in the Included Studies (Table 4)

All 16 trials were randomised trials; however, explicit generation of the allocation sequence was described in 6 trials or (by their authors during telephone calls) [16, 18, 20, 22, 23, 28] in which a random number generator was used. Four trials explicitly described adequate allocation concealment. In these trials, the allocation sequence was concealed by an opaque envelope [18, 23] or administered by a third party who was not involved in the study [16, 22]. The authors of two other trials [16, 22] did not describe allocation concealment clearly.

None of the included trials were confirmed to use blinding. This may have been due to the difficulty in preparing of placebo, which is similar to *Ginkgo biloba *extract, with the same appearance and feeling during injection. 

After we telephoned authors, a bias of incomplete outcome data was reported in 4 trials [18, 22, 23, 28]. One trial was confirmed to have incomplete outcome data [18]. 4 trials [16, 18, 22, 23] did not have selective reporting.

### 3.2. Effects of Interventions

#### 3.2.1. UAER

We analysed the effect of *Gingko biloba* extract on UAER in two groups according to different interventions: group 1 (*Gingko biloba* extract plus conventional treatment versus conventional treatment only) and group 2 (*Gingko biloba* extract combined with ACEi/ARB versus ACEi/ARB alone). 

**Figure 2:**
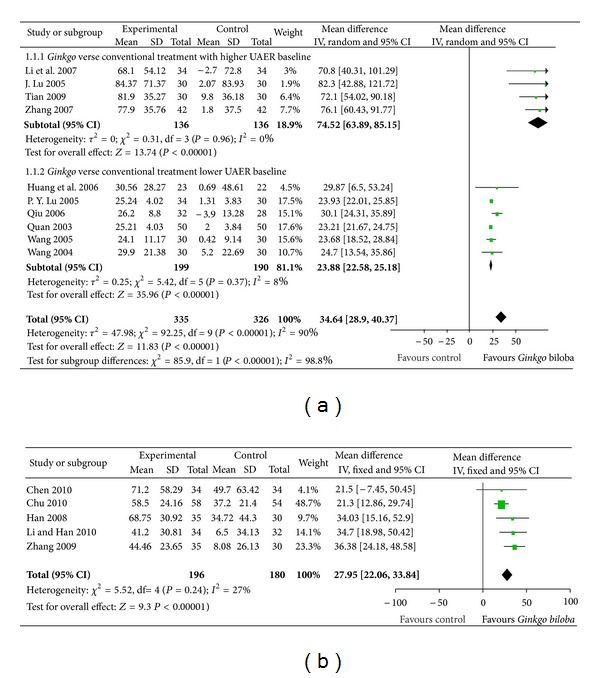
Comparison 1. Urinary albumin excretion ratio (UAER): (a) *Ginkgo* added to conventional treatment versus conventional treatment alone, (b) ginkgo combined with ACEi or ARB versus ACEi/ARB alone.

For Group 1, the UAER data were available in 10 trials with a total sample size of 661 participants. At the end of the study, UAER decreased with an overall effect size of 34.64 *μ*g/min (95% CI, from 28.90 to 40.37, *P* < 0.00001) (Figure 2(a)), in favour of the *Gingko biloba* extract group using a random-effects model. The test for heterogeneity in the total group indicated an *I* ^2^ value of 90%. This significant heterogeneity could be explained by the different UAER baseline levels of the patients enrolled in these trials. We then divided group 1 into two subgroups based on baseline UAER levels.

Subgroup 1.1.1 (Figure 2(a)) had a UAER baseline of >150 *μ*g/min and a higher effect size of 74.52 *μ*g/min (from 63.89 to 85.15, *P* < 0.00001) with no significant heterogeneity in the analysis. Subgroup 1.1.2 (Figure 2(a)) had a UAER baseline <100 *μ*g/min and lower effect size of 23.88 *μ*g/min (from 22.58 to 25.18, *P* < 0.00001) with low heterogeneity in the analysis.

The role of different baseline UAER levels was tested by metaregression using STATA 11.0 SE. The results showed a significant influence of the covariables (Table 1).

For Group 2, three trials including 376 participants reported urea levels, which decreased with an overall effect size of 27.95 *μ*g/min (from 22.06 to 33.84, *P* < 0.00001) (Figure 2(b)).

#### 3.2.2. Blood Glucose

**Figure 3:**
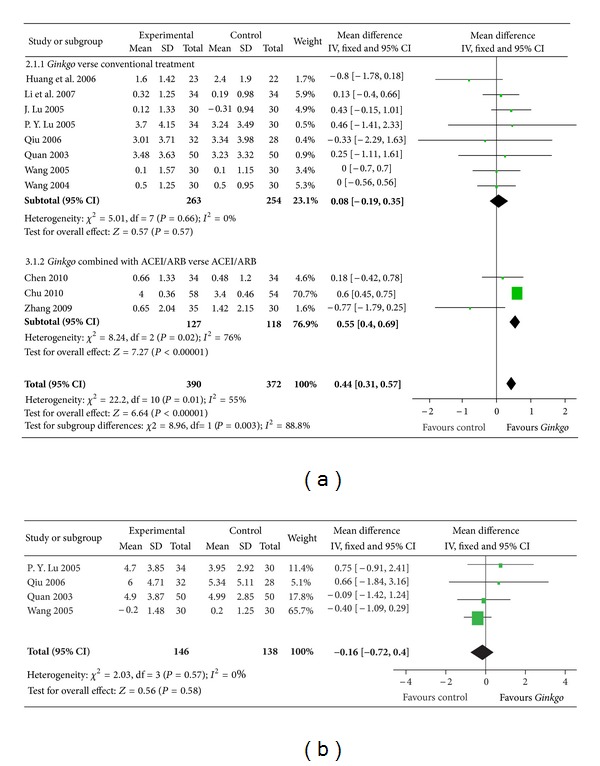
Comparison 2. Blood glucose: (a) fasting blood-glucose (FBG), (b) postprandial blood glucose (PBG).

Data for FBG were presented in 11 trials with a total of 762 participants (Figure 3(a)). FBG decreased with an overall effect size of 0.44 mmol/L (0.31 to 0.57, *P* < 0.00001) with a fixed-effects model in favour of the *Gingko biloba* extract group. 

PBG was presented in four trials with a total of 284 participants (Figure 3(b)). PBG decreased with an overall effect size of −0.16 mmol/L (from −0.72 to 0.40, *P* = 0.58), which was not statistically significant compared to the control group.

#### 3.2.3. Kidney Function

**Figure 4:**
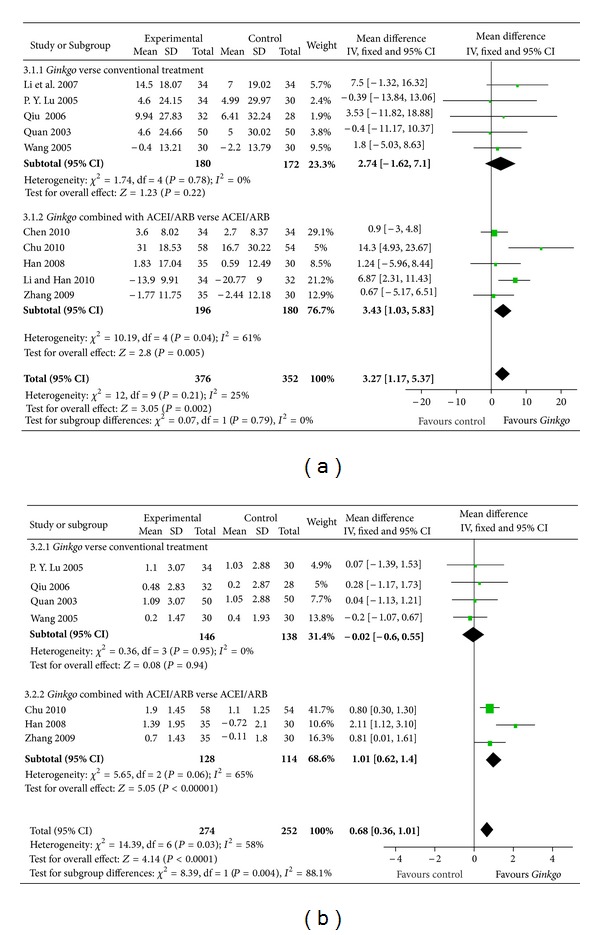
Comparison 3. Kidney function: (a) serum creatinine (SCR), (b) blood urea nitrogen (BUN).

SCR data were presented in 10 trials with a total of 728 participants (Figure 4(a)). SCR decreased with an overall effect size of 3.27 *μ*mol/L (from 1.17 to 5.37, *P* = 0.002) with a fixed-effects model in favour of the *Gingko biloba* group. But no statistically significant effect in SCR was observed in subgroup 3.1.1 (*P* = 0.22) (Figure 4(a)).

Seven trials including 526 participants were analysed. BUN decreased by 0.68 mmol/L compared to the control group (from 0.36 to 1.01, *P* < 0.00001; Figure 4(b)). But in the subgroup analysis, BUN increased by 0.02 mmol/L, which was not statistically significant compared to the control group (from 0.62 to 1.40, *P* = 0.06; Figure 4(b)).

#### 3.2.4. Hemorrheology

**Figure 5:**
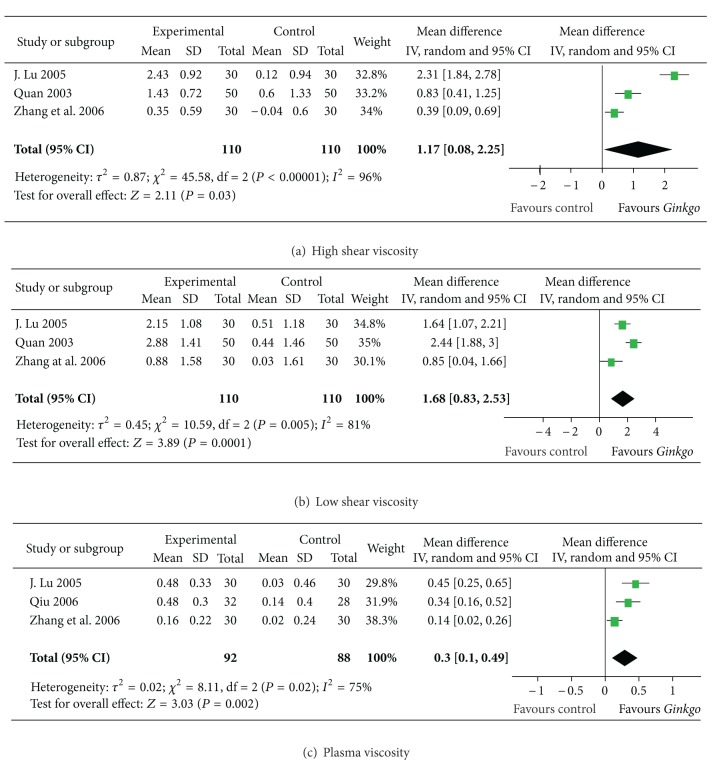
Comparison 6. Hemorrheology: (a) high-cut whole blood viscosity, (b) low-cut whole blood viscosity, and (c) plasma viscosity.

In three trials with 220 participants, high shear viscosity and low shear viscosity were presented. The former decreased with an overall effect size of 1.17 mPa·s (from 0.08 to 2.25, *P* = 0.003; Figure 5(a)), and the latter decreased with an overall effect size of 1.68 mPa·s (from 0.83 to 2.53, *P* = 0.005; Figure 5(b)). Plasma viscosity was presented in three trials, with a total of 180 participants. Plasma viscosity decreased by 0.30 mPa·s compared to controls (from 0.10 to 0.49, *P* = 0.002; Figure 5(c)).

#### 3.2.5. Adverse Effects

No study reported serious adverse effects such as bleeding [8]. Mild cough was reported in only one trial [19], in which the intervention was combined with ACEi. 2 trials [22, 23] reported no adverse effects in patients during observation. The other 13 trials did not report whether adverse effects were observed.

#### 3.2.6. Publication Bias

**Figure 6:**
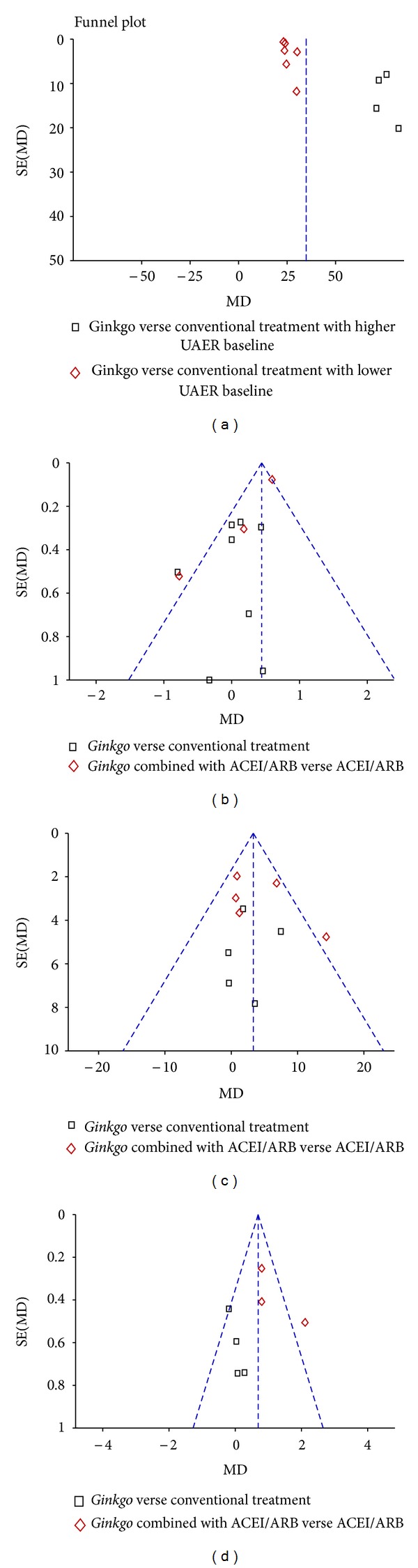
Funnel plot comparison. (a) Urinary albumin excretion rate (UAER) with ginkgo add-on conventional treatment versus conventional treatment alone, (b) fasting blood glucose (FBG), (c) serum creatinine (SCR), (d) blood urea nitrogen (BUN).

The funnel plots of FBG and BUN (Figure 6) appear asymmetric, suggesting evidence of publication bias. No publication bias was observed form the funnel plots of UAER and SCR.

## 4. Discussion

### 4.1. Summary of Findings

The principal finding of this paper is that *Gingko biloba* extract may be beneficial for early diabetic nephropathy by decreasing the UAER, lowering FPG, and improving kidney function and the hemorrheology outcome measurements. 

Microalbuminuria is associated with an increased likelihood of progression of generic chronic kidney disease (CKD) to more advanced stages or even to ESRD [35–38], and increased albumin exertion, even below the lower limit of conventional microalbuminuria, is associated with an increased likelihood for both all cause and CV-related mortality in patients with CKD [39]. Hence, the level of microalbuminuria has more of an impact on renal endpoints (now defined as the need to start RRT) than the level of GFR per se in CKD stages from 1 to 3 [40, 41].

ACEi and ARB reduce urinary albumin excretion and slow the progression of CKD [42]. However, new therapies to improve the prognosis of diabetic nephropathy are still needed. Microalbuminuria is a sign of systemic endothelial dysfunction, which may impair tubular epithelial albumin reabsorption. Extracts of *Gingko biloba* have been used to protect the vasculature of patients with diabetes [43, 44].

Our paper showed that *Gingko biloba* extract decreased UAER level during early diabetic nephropathy. In addition, the baseline UAER level may influence the effect of *Gingko biloba* according to metaregression analysis. UAER with a high baseline level >150 *μ*g/min was decreased by 74.52 *μ*g/min; however, UAER with a low level baseline of <100 *μ*g/min was decreased by 23.88 *μ*g/min. The effect of *Gingko biloba* may be more remarkable with a high level of pretreatment. However, more studies are needed to confirm the relationship between baseline UAER levels and the effect of *Gingko biloba*.

Abnormal hemodynamic indexes, such as increase in whole blood viscosity and plasma viscosity, are risk factors for development of diabetic nephropathy [45–47]. Our paper showed that *Gingko biloba* extract decreased blood viscosity and plasma viscosity in patients with early diabetic nephropathy. One study [48] conducted in Taipei also confirmed the effect of *Gingko biloba* extract on hemorheological indices in patients with diabetic retinopathy which are also microvascular complication of diabetes. Molecular biological mechanism of hemorheological disturbance in diabetes is not very clear. It may be associated with lipid peroxidation stress or reduced antioxidant vitamin E content of cell membranes [48]. Although experiments have demonstrated that *Gingko biloba* has the effect of antioxidation [49, 50], further studies about the effect and mechanism of *Gingko biloba *on hemorrheology are still needed.

In all trials included in our paper, kidney function was reported as SCR and BUN. Well-controlled UAER and improved haemodynamics play important roles in kidney protection. However, the effects of extract on SCR and BUN found in our subgroup analysis were not statistically significant, although the pooled effect size of the entire group had statistical significance. It was possibly due to the short course (from 14 to 60 days) of treatment in the included trials.

*Ginkgo biloba* extract may influence BG control by decreasing the effect of oral glucose-lowering drugs [51]. However, a series of clinical randomised studies has shown that *Gingko biloba* extract decreases BG by increasing pancreatic *β*-cell function [52, 53] without producing insulin resistance in patients with type 2 diabetes with pancreatic exhaustion [54]. In our analysis, *Gingko biloba *had effect on lowering FBG, but no obvious influence on PBG.

### 4.2. Safety Assessment

As some studies reported, *Gingko biloba* extract could increase the risk of bleeding [8], such as intracranial hemorrhage or hyphema [55, 56]. These reports were mainly case reports, with dose varying from 100 mg to 240 mg daily and durations from 4 weeks to 52 weeks. And *Gingko biloba* extract in these reports was often used with anticoagulants or after surgery. In this paper, no one reported bleeding with lower dose (between 35 mg to 70 mg daily) and shorter duration (between 2 weeks to 9 weeks). But most studies included in this paper did not report whether they made observation on adverse events. The safety of *Gingko biloba* extract for early diabetic nephropathy still needs further research to estimate if there are any side effect.

In addition, several clinical studies observed herb drug interactions between *Ginkgo biloba* extract and conventional medicinals used in early diabetic nephropathy, such as tolbutamide [57, 58], talinolol [59, 60], metformin [61], nifedipine [62], and atorvastatin [63]. It was reported that combined use of *Ginkgo biloba* extract with tolbutamide [57] or talinolol [59] might affect their efficacy at higher doses (between 320 mg daily and 360 mg daily, resp.). In this review, no study reported herb drug interaction caused by *Ginkgo biloba* extract. May be it is due to the lower doses (from 35 mg to 70 mg daily) of *Ginkgo biloba* extract used in all the included trials. However, there is still absence of evidence about the influence of *Ginkgo biloba* extract on the efficacy of conventional medicinals. Further investigation of herb drug interaction with conventional treatment should be undertaken.

### 4.3. Overall Completeness and Applicability of Evidence

Our findings are generalisable to the majority of patients with early diabetic nephropathy being treated with *Gingko biloba* extract added to conventional medicine or ACEi/ARB. It is possible that the effect of *Gingko biloba* extract on decreasing microalbuminuria will be stronger at the high baseline level. However, additional RCTs are needed to confirm this.

### 4.4. Quality of the Evidence

Although 16 trials were included in our paper, randomisation methods were elaborated in 6 trials. We confirmed that none of the trials were blinded, which could have affected the results. Some doctors might pay more attention to patients in a treatment group than those in a control group. In addition, some of the studies were conducted several years ago, and the authors may have forgotten the details of some incomplete outcomes, which could have resulted in selective reporting.

### 4.5. Potential Biases in the Paper Process

Variation among trials was apparent in terms of sample size, treatment course, dosage, and forms of *Gingko biloba* extract used. Extract was used in combination with ACEi/ARB in several studies. However, the effects of *Gingko biloba* extract alone, *Gingko biloba* extract combined with ACEi, and *Gingko biloba* extract combined with ARB could not be clarified on some outcome measurements due to the small number of studies.

Conventional therapy including control of glucose, lipid, and BP is necessary as a general intervention according to the guidelines. However, this conventional therapy was not be well reported in the included studies, and we were unable to adjust for potential influences in our analyses.

The statistical heterogeneity among trials in this study was apparently substantial. Heterogeneity in UAER, FBG, and BUN mainly originates from subgroups allocated according to different baseline values or interventions. Heterogeneity in hemorrheology may be explained by differences in baseline characteristics among participants.

Although a series of comprehensive search strategies were conducted, the results of our paper were based on published studies. The funnel plots of FBG and BUN appear asymmetric, suggesting evidence of publication bias in the literature. But poor methodological quality in smaller studies and heterogeneity due to different interventions should also be considered as possible reasons for asymmetry.

## 5. Conclusion 

*Gingko biloba* extract is a valuable drug which has prospect in treating early diabetic nephropathy, as it may decrease UAER and FBG and improve hemorrheology. Patients with a higher baseline UAER level may gain a better effect from *Gingko biloba* extract. The safety of *Gingko biloba* extract for early diabetic nephropathy is still uncertain. Herb drug interactions between *Ginkgo biloba *extract and conventional medicinals need further investigation to indicate. Nevertheless, the methodological quality in included studies was not perfect, and there were potential biases due to different interventions and short courses of treatment that may have affected the results. Long-term, double-blinded RCTs with large sample sizes are still needed to provide stronger evidence.

## Figures and Tables

**Table 1:** Result of the metaregression.

	Number of studies	Coefficient (95% CI)	*t*	*P*	Tau^2^	*I* ^2^ Res (%)	Adjusted *R* ^2^ (%)
Urea baseline	10	−50.74 (−63.32, −38.16)	−9.30	<0.001	0.00	0.00	100.00

**Table 2:** Characteristics of the included studies.

Author, year	Study design	Duration	No. of participants, treatment group/control group	Treatment group: Age (yrs) mean, SD or min-max	Control group: Age (yrs) mean, SD or min-max	Total group: Age (yrs) mean, SD or min-max	Treatment group: Medication, name, form, dosage	Control group: Medication, name, form, dosage	Adverse events
Chen, 2010 [15]	RCT, Ginkgo + ACEi/ARB versus ACEi/ARB	21 days	34/34	44, 6	43, 8	Unclear	*Ginkgo biloba* extract injection 20 mL qd, Erbesartan oral 150 mg qd	Erbesartan oral 150 mg qd	Not noted

Chu, 2010 [16]	RCT, Ginkgo + ACEi/ARB versus ACEi/ARB	56 days	58/54	56.1, 14.4	56.2, 15.6	Unclear	*Ginkgo biloba* extract injection 20 mL qd, valsartan 80 mg qd	Valsartan 80 mg qd	Not noted

Han, 2008 [17]	RCT, Ginkgo + ACEi/ARB versus ACEi/ARB	42 days	35/30	Unclear	Unclear	50.6, 12.5	*Ginkgo biloba* extract injection 20 mL qd, Lotensin 10–20 mg qd	Lotensin 10–20 mg qd	Not noted

Huang et al., 2006 [18]	RCT, Ginkgo + conventional treatment versus conventional treatment	14 days	23/22	54, 9	54, 9	Unclear	*Ginkgo biloba* extract injection 20 mL qd	Conventional treatment	Not noted

Li and Han, 2010 [19]	RCT, Ginkgo + ACEi/ARB versus ACEi/ARB	28 days	36/32	Unclear	Unclear	46–74	*Ginkgo biloba* extract injection 20 mL qd, Fosinopril oral 10 mg qd	Fosinopril oral 10 mg qd	Mild cough three in treatment group, two in control group

Li et al., 2007 [20]	RCT, Ginkgo + conventional treatment versus conventional treatment	60 days	34/29	66.19, 7.13	68.2, 71.7	Unclear	*Ginkgo biloba* extract 2 tables tid	Conventional treatment	Not noted

P. Y. Lu, 2005 [21]	RCT, Ginkgo + conventional treatment versus conventional treatment	28 days	34/30	41–78	45–72	Unclear	*Ginkgo biloba* extract injection 20 mL qd	Conventional treatment	Not noted

J. Lu, 2005 [22]	RCT, Ginkgo + conventional treatment versus conventional treatment	28 days	30/30	58.9, 8.5	57.1, 7.9	Unclear	*Ginkgo biloba* extract injection 15 mL qd	Conventional treatment	No adverse effect was found

Qiu, 2006 [23]	RCT, Ginkgo + conventional treatment versus conventional treatment	14 days	32/28	Unclear	Unclear	51, 9	*Ginkgo biloba* extract injection 20 mL qd	Conventional treatment	No adverse effect was found

Quan, 2003 [24]	RCT, Ginkgo + conventional treatment versus conventional treatment	28 days	50/50	Unclear	Unclear	50.5, 12.5	*Ginkgo biloba* extract injection 20 mL qd	Conventional treatment	Not noted

Tian, 2009 [25]	RCT, Ginkgo + conventional treatment versus conventional treatment	28 days	30/30	59.8, 6.4	60.3, 5.7	Unclear	*Ginkgo biloba* extract injection 20 mL qd	Conventional treatment	Not noted

Wang, 2003 [26]	RCT, Ginkgo + conventional treatment versus conventional treatment	28 days	30/30	54.3, 5.2	56.0, 6.5	Unclear	*Ginkgo biloba* extract injection 20 mL qd	Conventional treatment	Not noted

Wang, 2005 [27]	RCT, Ginkgo + conventional treatment versus conventional treatment	28 days	30/30	36.7, 11.3	37.1, 10.9	Unclear	*Ginkgo biloba* extract injection 20 mL qd	Conventional treatment	Not noted

Zhang, 2009 [28]	RCT, Ginkgo + ACEi/ARB versus ACEi/ARB	28 days	35/30	Unclear	Unclear	50.2, 8.5	*Ginkgo biloba* extract injection 20 mL qd, Benazepril 10 mg qd	Benazepril 10 mg qd	Not noted

Zhang, 2007 [29]	RCT, Ginkgo + conventional treatment versus conventional treatment	21 days	42/42	58.2, 3.6	59.5, 3.4	Unclear	*Ginkgo biloba* extract injection 10 mL qd	Conventional treatment	Not noted

Zhang et al., 2006 [30]	RCT, Ginkgo + conventional treatment versus conventional treatment	20 days	30/30	Unclear	Unclear	51.2, 2.3	*Ginkgo biloba* extract injection 20 mL qd	Conventional treatment	Not noted

RCT: random control trial; ACEi: angiotensin-converting enzyme inhibitor; ARB: angiotensin II receptor blockers.

**Table 3:** Numerical data of outcomes of the included studies.

	Outcomes measured
Author, year	Numerical data of outcomes (difference before and after treatment; mean, SD)
	Treatment group/control group
Chen, 2010 [15]	Outcomes measured: UAER, FBG, SCR UAER: −71.2, 58.29/−49.7, 63.42; FBG: −0.66, 1.33/−0.48, 1.2; SCR: −3.6, 8.02/−2.7, 8.37
Chu, 2010 [16]	Outcomes measured: UAER, FBG, BUN, SCR UAER: −58.5, 24.16/−37.2, 21.4; FBG: −4, 0.36/−3.4, 0.46; BUN: −1.9, 1.45/−1.1, 1.25; SCR: −31, 18.53/−16.7, 30.22
Han, 2008 [17]	Outcomes measured: UAER, SCR, BUN UAER: −68.75, 30.92/−34.72, 44.30; SCR: −1.83, 17.04/0.59, 12.49; BUN: −1.39, 1.95/0.72, 2.10
Huang et al., 2006 [18]	Outcomes measured: UAER, FBG UAER: −30.56, 28.27/−0.69, 48.61; FBG: −1.6, 1.42/−2.4, 2.12
Li and Han, 2010 [19]	Outcomes measured: UAER, SCR UAER: −41.2, 30.81/−6.5, 34.13; SCR: 13.9, 9.91/20.77, 9.0
Li et al., 2007 [20]	Outcomes measured: UAER, SCR, FBG UAER: −68.1, 54.12/2.7, 72.8; SCR: −14.5, 18.07/−7, 19.02; FBG: −0.32, 1.25/−0.19, 0.98
P. Y. Lu, 2005 [21]	Outcomes measured: UAER, SCR, BUN, FBG, PBG UAER: −25.24, 4.02/−1.31, 3.83; SCR: −4.6, 24.15/−4.99, 29.97; BUN: −1.1, 3.07/−1.03, 2.88; FBG: −3.7, 4.15/−3.24, 3.49; PBG: −4.7, 3.85/−3.95, 2.92
J. Lu, 2005 [22]	Outcomes measured: UAER, FBG, high shear viscosity, low shear viscosity, plasma viscosity UAER: −84.37, 71.37/−2.07, 83.93; FBG: −0.12, 1.33/0.31, 0.94; high shear viscosity: −2.43, 0.92/−0.12, 0.94; low shear viscosity: −2.15, 1.08/−0.51, 1.18, plasma viscosity: −0.48, 0.33/−0.03, 0.46.
Qiu, 2006 [23]	Outcomes measured: UAER, SCR, BUN, FBG, PBG, plasma viscosity UAER: −26.20, 8.80/3.90, 13.28; SCR: −9.94, 27.83/−6.41, 32.24; BUN: −0.48, 2.83/−0.2, 2.87; FBG: −3.01, 3.71/−3.34, 3.98; PBG: −6, 4.71/−5.34, 5.11, plasma viscosity: −0.48, 0.30/−0.14, 0.40.
Quan, 2003 [24]	Outcomes measured: UAER, SCR, BUN, FBG, PBG, low shear viscosity, high shear viscosity UAER: −25.21, 4.03/−2.00, 3.84, SCR: −4.6, 24.66/−5, 30.02; BUN: −1.09, 3.07/−1.05, 2.88; FBG: −3.48, 3.63/−3.23, 3.32; PBG: −4.9, 3.87/−4.99, 2.85; low shear viscosity: −2.88, 1.41/−0.44, 1.46; high shear viscosity: −1.43, 0.72/−0.6, 1.33
Tian, 2009 [25]	Outcome measured: UAER UAER: −81.9, 35.27/−9.8, 36.18
Wang, 2005 [26]	Outcomes measured: UAER, SCR, BUN, FBG, PBG UAER: −24.1, 11.17/0.42, 9.14; SCR: 0.4, 13.21/2.2, 13.79; BUN: −0.2, 1.47/−0.4, 1.93; FBG: −0.1, 1.57/−0.1, 1.15; PBG: 0.2, 1.48/−0.2, 1.25
Wang, 2004 [27]	Outcomes measured: UAER, FBG UAER: −29.9, 21.38/−5.2, 22.69; FBG: −0.5, 1.25/−0.5, 0.95
Zhang, 2009 [28]	Outcomes measured: UAER, SCR, BUN, FBG UAER: −44.46, 23.65/−8.08, 26.13; SCR: 1.77, 11.75/2.44, 12.18; BUN: −0.7, 1.43/0.11, 1.80; FBG: −0.65, 2.04/−1.42, 2.15
Zhang, 2007 [29]	Outcomes measured: UAER, FBG UAER: −77.9, 35.76/−1.8, 37.5; FBG: −2.22, 1.23/−2.14, 1.20
Zhang et al., 2006 [30]	Outcomes measured: low shear viscosity, high shear viscosity, plasma viscosity Low shear viscosity: −0.88, 1.58/−0.03, 1.61; high shear viscosity: −0.35, 0.59/−0.04, 0.60; plasma viscosity: −0.16, 0.22/−0.02, 0.24.

UAER: urinary albumin excretion ratio; SCR: serum creatinine; BUN: blood urea nitrogen; FBG: fasting blood-glucose; PBG: postprandial blood gluco.

**Table 4:** Methodological quality of analysed studies.

Author, year	Random sequence generation	Allocation concealment	Blinding	Incomplete outcome data	Selective reporting	Other bias
Chen, 2010 [15]	Unclear	Unclear	High risk	Unclear	Unclear	Unclear
Chu, 2010 [16]	Low risk	Low risk	High risk	Unclear	Low risk	Unclear
Han, 2008 [17]	Unclear	Unclear	High risk	Unclear	Unclear	Unclear
Huang et al., 2006 [18]	Low risk	Low risk	High risk	High risk	Low risk	Unclear
Li and Han, 2010 [19]	Unclear	Unclear	High risk	Unclear	Unclear	Unclear
Li et al., 2007 [20]	Low risk	Unclear	High risk	Unclear	Unclear	Unclear
P. Y. Lu, 2005 [21]	Unclear	Unclear	High risk	Unclear	Unclear	Unclear
J. Lu, 2005 [22]	Low risk	Low risk	High risk	Low risk	Low risk	Unclear
Qiu, 2006 [23]	Low risk	Low risk	High risk	Low risk	Low risk	Unclear
Quan, 2003 [24]	Unclear	Unclear	High risk	Unclear	Unclear	Unclear
Tian, 2009 [25]	Unclear	Unclear	High risk	Unclear	Unclear	Unclear
Wang, 2005 [26]	Unclear	Unclear	High risk	Unclear	Unclear	Unclear
Wang, 2004 [27]	Unclear	Unclear	High risk	Unclear	Unclear	Unclear
Zhang, 2009 [28]	Low risk	Unclear	High risk	Low risk	Unclear	Unclear
Zhang, 2007 [29]	Unclear	Unclear	High risk	Unclear	Unclear	Unclear
Zhang et al., 2006 [30]	Unclear	Unclear	High risk	Unclear	Unclear	Unclear

## Appendix

### A. Search Strategies

#### A.1. Pubmed Database

#1 Nephropathy, Diabetic OR Nephropathies, Diabetic OR Diabetic Nephropathy OR Diabetic Kidney Diseases OR Kidney Disease, Diabetic OR Kidney Diseases, Diabetic OR Diabetic Glomerulosclerosis OR Kimmelstiel-Wilson Syndrome OR Kimmelstiel Wilson Syndrome OR Syndrome, Kimmelstiel-Wilson OR Kimmelstiel-Wilson Disease OR Kimmelstiel Wilson Disease OR Nodular Glomerulosclerosis OR Glomerulosclerosis, Nodular OR Glomerulosclerosis, Diabetic OR Intracapillary Glomerulosclerosis

#2 Traditional Chinese Medicine OR Chinese Traditional Medicine OR Chinese Herbal Drugs OR Chinese Drugs, Plant OR Medicine, Traditional OR Ethnopharmacology OR Ethnomedicine OR Ethnobotany OR Medicine, Kampo OR Kanpo OR TCM OR T.C.M. OR Medicine, Ayurvedic OR Alternative Medicine OR Complementary Medicine OR Phytotherapy OR Herbology OR Plants, Medicinal OR Plant Preparations OR Plant Extracts OR Plants, Medicine OR Materia Medica OR Single Prescription OR Acupuncture OR Meridians OR Electroacupuncture OR Moxibustion OR Auriculotherapy OR Catgut embedding OR Herbs OR Chinese Medicine Herb OR Herbal Medicine.

#3 Clinical Trial OR clinical study OR Controlled Trial OR Controlled study OR random* control* Trial OR random* control* study OR Multicenter Study OR Meta-Analysis OR placebo control OR dummy control OR blinding OR clinical research OR medical trial

#4 #1 AND #2 AND #3

#5 Search “*Ginkgo biloba*”

#6 Search “Egb 761”

#7 Search “Tavonin”

#8 Search “tavonin”

#9 Search “tebonin”

#10 Search “Rokan”

#11 Search “tanakan”

#12 Search “LI 1370”

#13 Search “EGB”

#14 Search “bilobalid”

#15 Search “kaveri”

#16 Search ginkgo$ OR gingko$ OR gingko OR ginko$ OR gingko OR gincosan

#17 #5 OR #6 OR #7 OR #8 OR #9 OR #10 OR #11 OR #12 OR #13 OR #14 OR #15 OR #16

#18 #1 and #17 Limits: Humans, Clinical Trial, Meta-Analysis, Randomised Controlled Trial, Review

#19 #4 OR #18

#### A.2. Embase Database

*Advanced Search.* #1 Nephropathy, Diabetic OR Nephropathies, Diabetic OR Diabetic Nephropathy OR Diabetic Kidney Diseases OR Kidney Disease, Diabetic OR Kidney Diseases, Diabetic OR Diabetic Glomerulosclerosis OR Kimmelstiel-Wilson Syndrome OR Kimmelstiel Wilson Syndrome OR Syndrome, Kimmelstiel-Wilson OR Kimmelstiel-Wilson Disease OR Kimmelstiel Wilson Disease OR Nodular Glomerulosclerosis OR Glomerulosclerosis, Nodular OR Glomerulosclerosis, Diabetic OR Intracapillary Glomerulosclerosis

#2 Diabetes nephropathy OR diabetic glomerulopathy OR diabetic glomerulosclerosis OR diabetic intercapillary glomerulosclerosis OR diabetic nephropathies OR diabetic nephrosclerosis OR glomerulonecrosis, intercapillary; glomerulosclerosis, diabetic OR glomerulosclerosis, intercapillary; intercapillary glomerulosclerosis OR Kimmelstiel Wilson syndrome OR kimmelstiel wilson disease OR kimmelstiel wilson nephropathy OR kimmelstiel wilson syndrome OR nephropathy, diabetic

#3 Traditional Chinese Medicine OR Chinese Traditional Medicine OR Chinese Herbal Drugs OR Chinese Drugs, Plant OR Medicine, Traditional OR Ethnopharmacology OR Ethnomedicine OR Ethnobotany OR Medicine, Kampo OR Kanpo OR TCM OR T.C.M. OR Medicine, Ayurvedic OR Alternative Medicine OR Complementary Medicine OR Phytotherapy OR Herbology OR Plants, Medicinal OR Plant Preparations OR Plant Extracts OR Plants, Medicine OR Materia Medica OR Single Prescription OR Acupuncture OR Meridians OR Electroacupuncture OR Moxibustion OR Auriculotherapy OR Catgut embedding OR Herbs OR Chinese Medicine Herb OR Herbal Medicine

#4 #1 or #2

#5 #4 and #3 Quick limits “humans,” limit “MJ,” advanced limits “RCT,” “CT”

#6 “*ginkgo biloba*”/exp OR “egb 761”/exp OR “tavonin” OR “tebonin”/exp OR “rokan”/exp OR “tanakan”/exp OR “li 1370”/exp OR “egb” OR “bilobalid” OR “kaveri”/exp OR ginkgo$ OR gingko$ OR ginko$ OR gingko OR gincosan

#7 #4 and #6 Quick limits “humans,” limit “MJ,” advanced limits “RCT,” “CT”

#8 #5 OR #7

*Disease Search*

#9 # 3 OR #6

Subject: diabetic nephropathy

Subheading:

Disease management

Drug therapy

Prevention

Rehabilitation

Therapy

Quick limits “humans” Advanced limits “RCT,” “CT”.
